# The School Clinic

**Published:** 1911-11-25

**Authors:** 


### THE SCHOOL CLINIC.

### IV.—The Educative Value of the Clinic.

The article on School Clinics from a general practitioner's point of view suggests a few remarks. It is stated that "the educative value of inspection is not yet fully realised by most of us." The word "educative" appears to be used by one who seems to have some doubt regarding the usefulness of statistics gained through medical inspection, yet who, on the whole, considers that they will eventually add to our knowledge of medicine. The word "educative," however, used in connection with medical inspection may have several meanings. It may apply to instruction to parents in the need for attention to the health of their children; to knowledge gained by the inspector himself in his examination of children; or, in the way suggested above, to the general knowledge gained by means of statistics. Statistics are proverbially fallacious, and they are fallacious owing to the unfortunate fallibility of human observation and judgment. The educational influence of medical inspection so far as the parents of school-children are concerned is obvious. There is, however, one aspect of the educative value of medical inspection which has to some extent been lost sight of. This is the educative value to the inspector himself.

### The Education of the Inspector.

It is to be feared that as medical inspection is at present organised the educative value of inspection to the inspectors themselves is not great. Unless they have had considerable experience of diseases of children before they take up school inspection, the addition to their knowledge gained by inspection in schools will not be great. If they start with a mistaken idea regarding some condition, they have no means by which that erroneous idea may be corrected. In any doubtful case they cannot appeal to the senior school officer, becase he is a medical officer of health who will not pretend to know anything material about children's diseases. To use a simple illustration. It is not uncommon in examination of children to find a variety of intermittence of the pulse. The writer of this article has known several instances in which medical inspectors have diagnosed cardiac disease in children from this sign alone. If a medical inspector starts with the view that such a sign is diagnostic of cardiac disease there is nothing in the present system to prevent his maintaining such a view throughout life.

This question of heart affections might be enlarged upon. It is extraordinary how many mistakes are made over small everyday variations from what is considered the normal. Students are taught mainly from serious cases at the bedside, and the study of medicine from this limited field is apt to distort the vision. Strangely enough too, the teacher's vision may apparently also become distorted. For example, it was once stated to the writer that a teacher of medicine had been accustomed to say that a diastolic cardiac sound is an invariable sign of mitral stenosis, and a writer in one of the medical journals expressed a similar opinion. If this were true, the number of children with cardiac disease would indeed be great. Not only is this sound common in the absence of cardiac disease but systolic murmurs of no serious significance are frequent. Yet these murmurs are commonly considered, even by highly qualified medical inspectors, as diagnostic of disease of the heart. Such a diagnosis when wrong is serious, since it may materially affect the after-life of the child. Again, there is the question of tuberculous disease of the lungs, concerning which there has been much discussion. With regard to this point the views of inspectors are apparently becoming more reasonable.

### The Need for Consultation.

At one time it seemed as if the number of children to be discovered with chronic tuberculous disease of the lungs would be very great. Special knowledge may also sometimes be required in the diagnosis of nervous affections, such as some forms of spasmodic tic, or in the recognition of hysteria or of certain varieties of epilepsy. A case once came under the writer's notice in which hysterical œdema and hæmorrhage followed a slight blow on the back of the hand of a girl in school. The girl is now herself a school teacher, and the swelling and hæmorrhage still occur from time to time. In connection with epilepsy the strange manifestations of this disease are so well-known that it is perhaps improbable that mistakes are likely to be made by inspectors though they may be by teachers. For example, a teacher was much puzzled by the occasional acts of an otherwise docile pupil who, in spite of protest, would sometimes leave her seat, go to a cupboard, and take out a book. Young medical men, however, often do not seem to realise that the great majority of "faints" in children are epileptic.

For medical inspection to be of educative value to the inspector he will need to have someone with whom to consult upon special cases. How this aid may best be provided is not perhaps easy to decide. Dr. Priestley in advocating the view that school clinics should be established and treatment not conducted by the medical inspectors themselves, recommended supervision of these clinics by a medical man of special experience connected with some hospital or by some medical man in practice who had had special experience of diseases of children. A medical man in practice, yet perhaps not in busy practice, who has been resident medical officer in a children's hospital, might be able to render much valuable service in the way of advice. We all have in some degree our special interests, and medical men in practice attached to clinics would like to have their special interests further stimulated by a request for an opinion upon a case belonging to some class of disease to which attention has been paid. Should medical inspection become too self-contained all concerned in it must suffer.

